# Two-port connecting-layer-based sandwiched grating by a polarization-independent design

**DOI:** 10.1038/s41598-017-01424-w

**Published:** 2017-05-02

**Authors:** Hongtao Li, Bo Wang

**Affiliations:** School of Physics and Optoelectronic Engineering, Guangdong University of Technology, Guangzhou, 510006 China

## Abstract

In this paper, a two-port connecting-layer-based sandwiched beam splitter grating with polarization-independent property is reported and designed. Such the grating can separate the transmission polarized light into two diffraction orders with equal energies, which can realize the nearly 50/50 output with good uniformity. For the given wavelength of 800 nm and period of 780 nm, a simplified modal method can design a optimal duty cycle and the estimation value of the grating depth can be calculated based on it. In order to obtain the precise grating parameters, a rigorous coupled-wave analysis can be employed to optimize grating parameters by seeking for the precise grating depth and the thickness of connecting layer. Based on the optimized design, a high-efficiency two-port output grating with the wideband performances can be gained. Even more important, diffraction efficiencies are calculated by using two analytical methods, which are proved to be coincided well with each other. Therefore, the grating is significant for practical optical photonic element in engineering.

## Introduction

Polarization-independent gratings are performed as the critical optical periodic devices, which can be applied in the energy harvesting, nonlinear optics^1^, solar cells^2^, writing fiber Bragg applications^3^, liquid crystal-polymer composite^4^, filters^5–10^. In the reported studies, two-port polarization-independent gratings are investigated, where characteristics of high efficiency and broad bandwidth were exhibited. Zhao *et al*.^11^ have successfully designed a 50/50 splitting ratio non-polarizing beam splitter fused-silica transmission grating based on the modal method, which can be proved to be realizable. For fabricating the conventional fused-silica grating, Feng *et al*.^12^ have successfully analyzed and optimized a two-port beam splitter grating, most importantly, they have fabricated the two-port fused-silica grating, which can be used the effective holographic interference and ICP etching technology. In addition, they researched a two-port beam splitter polarization-independent grating with a large grating groove depth, where the efficiencies in the 0th and the −1st orders were 47.31% and 47.42%, respectively for TE polarization. For fabricating the sandwiched grating, Clausnitzer *et al*.^13^ firstly cleaned the grating surface under the high-pressure environment and the grating activated by using the oxygen plasma. Next, a 5 kg weight of the fused silica can be pressed on the grating surface under 100 °C for three days. Lu *et al*.^14^ designed a unified two-port fused-silica grating, where the TE-polarized light diffracted into the 0th and the −1st orders with efficiencies of 47.4%. Wang *et al*.^15^ reported a binary phase two-port fused-silica polarization-independent grating, where the spectral widths of 40 nm was to be designed. According to a simplified modal method, a legible physical picture can be shown for not only gratings with simple structure but also for the complicated gratings^16–18^. In order to obtain the precise grating parameters, a rigorous coupled-wave analysis (RCWA) approach can give some exact computed results, therefore, some accurate grating parameters can be gained^19^. In addition, Metamaterials can also achieve similar functional effect by introducing the additional phases and then changing their transmittance response^20, 21^. As is well known, it is the first time that we report and design a new two-port connecting-layer-based sandwiched polarization-independent grating under Littrow mounting. In this paper, a two-port connecting-layer-based sandwiched polarization-independent grating is investigated. Based on the simplified modal method, the grating duty cycle is designed and the grating groove depth is estimated. Based on RCWA, the grating depth and thickness are optimized. In this design, we can obtain that the optimized diffraction efficiencies in the −1st order and the 0th order are 48.5% and 48.7%, respectively for TE polarization. In Ref. 12, the diffraction efficiencies in the −1st order and the 0th order are 47.42% and 47.31%, respectively for TE polarization. In Ref. 14, the TE-polarized wave can diffract into the −1st order with transmission efficiency of 47.4% and the 0th order with efficiency of 47.4%. Therefore, one can see that the improvement in the diffraction efficiencies for the −1st diffraction order and the 0th diffraction order can be exhibited in the paper compared to the refs 12 and 14, in addition, the presented grating groove depth is much lower, which can be more easily manufactured. Compared with Ref. 15, the studied grating spectral width of 63 nm is enhanced. For fabricating the presented sandwiched connecting-layer-based grating, the Ta_2_O_5_ substrate with a proper mask can be put into ICP system for manufacturing the optimized grating depth and grating connecting layer thickness, the fabrication process of the grating covering layer can be similar to the Clausnitzer’s^13^. In this work, we use a sandwiched grating to realize the two-port output splitting performance. Compared with the proposed surface-relief gratings, our reported sandwiched grating can achieve the higher diffraction efficiency for TE polarization and wider incident spectral bandwidth. According to investigate the cause of advantages, one can be seen that the Fresnel loss can be reduced by employing the cover layer^13, 18^. Consequently, the new design of the polarization-independent sandwiched grating would be good to put into use in vast semiconductor photoelectric devices.

## Results

**Figure 1:**
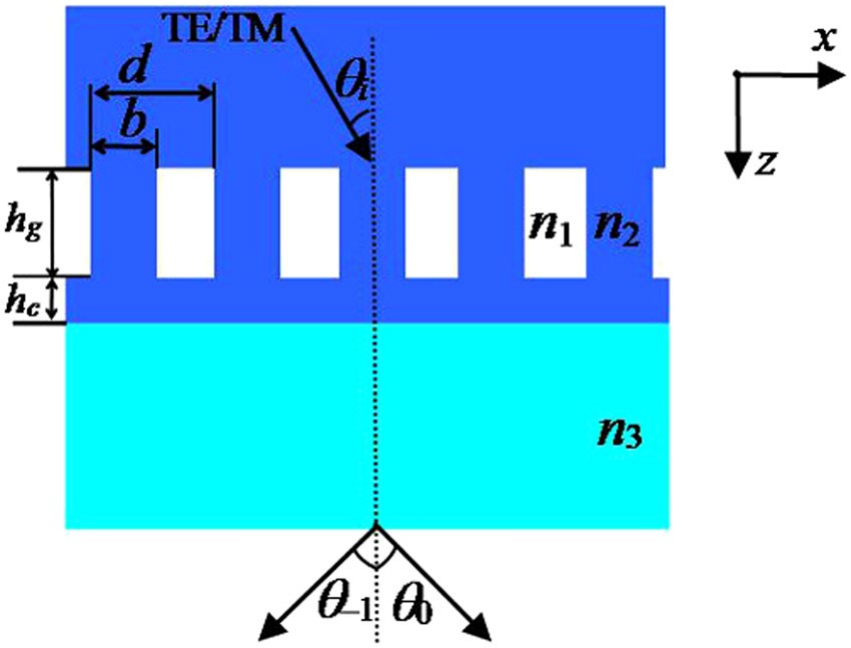
Schematic of a two-port sandwiched connecting-layer-based grating.

A two-port sandwiched connecting-layer-based grating with groove depth of *h*_*g*_ and connecting layer thickness of *h*_*c*_ is shown in Fig. 1. As can be seen from Fig. 1, under Littrow mounting, a polarized light can irradiate grating. In this condition, the incident angle can be called Littrow angle and the corresponding fixed equation is *θ*_*i*_ = sin^−1^(λ/2*n*_2_ *d*), where λ is incident wavelength, *n*_2_ indicates as the refractive indices of the covering layer and grating ridge and connecting layer, where *n*_2_ is about 1.45, and *d* represents as grating period. In addition, *n*_1_ = 1.0 and *n*_3 = _2.0 are the refractive indices of air and Ta_2_O_5_, respectively. The output angles of the −1st (*θ*_*−*1_) and the 0th (*θ*_0_) orders are the diffraction symmetric angles, which have been marked in Fig. 1. The proposed grating can diffract the polarized light in the −1st and the 0th orders, where the ability of energy distribution for two output diffraction orders are called the efficiencies.

**Figure 2:**
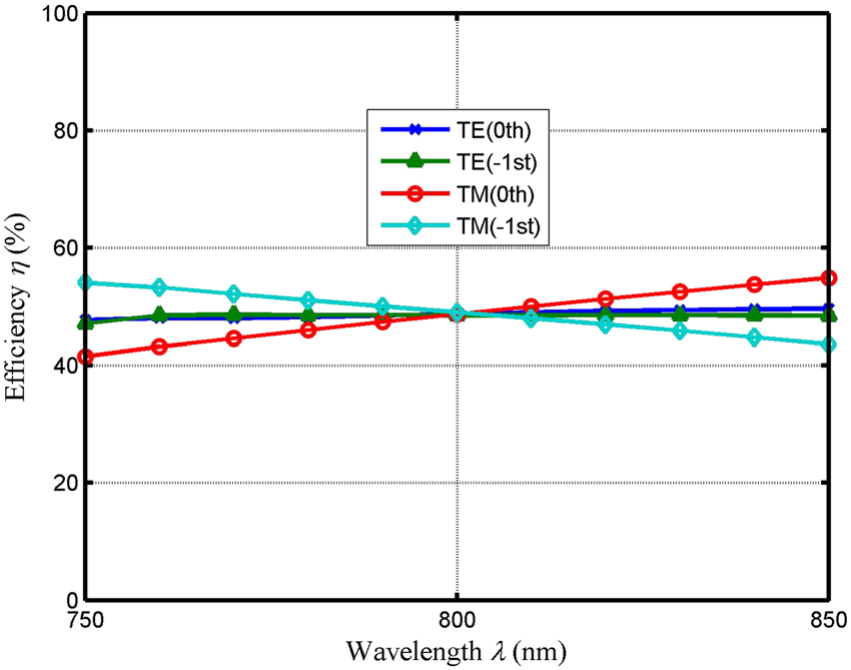
Efficiency versus the incident wavelength in the 0th and the −1st orders for both TE and TM polarizations at a duty cycle of 0.65 and an incident angle of 20.71°.

**Figure 3:**
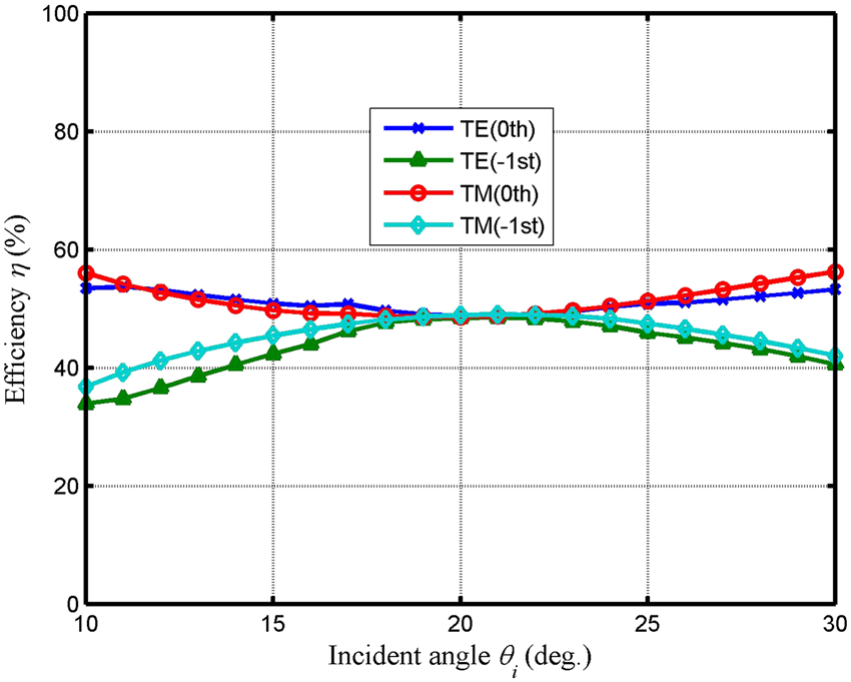
Efficiency versus the incident angle in the 0th order and the −1st order for both polarizations at a wavelength of 800 nm under the Littrow mounting.

During progress of the practical application, incident wavelength bandwidth (diffraction efficiencies in the −1st order and the 0th order above 45% versus the relative incident wavelength) and angular bandwidth (diffraction efficiencies in the −1st order and the 0th order above 45% versus the relative incident angle) are essential properties, which need to be taken into considered. In our design, in order to design the grating groove depth and the thickness of connecting layer, the grating wavelength is fixed, where its optimum value is 800 nm. The value of special duty cycle is the ratio of grating width to grating period, where the grating width is larger than the adjacent grating spacing in this paper. High efficiencies in each port for TE and TM polarizations are acquired, where the two bandwidths are deviated from the optimum grating wavelength of 800 nm and incident angle of 20.71°. Figure 2 reveals that the efficiency versus incident wavelength at a Littrow angle of 20.71° and special duty cycle of 0.65. As shown in the Fig. 2, efficiencies over than 45% in the −1st order and the 0th order are obtained within the wavelength range of 720–947 nm for TE-polarized light. Moreover, for TM-polarized light, efficiencies in the −1st and the 0th orders are more than 45%, where the incident band can be over an incident wavelength range of 774–837 nm. Figure 3 shows diffraction efficiency versus incident angle with the optimum groove depth and thickness. As shown in Fig. 3, efficiencies can be diffracted more than 45% into the −1st and the 0th orders in angle range of 16.57°−26.14° and 14.63°−27.57° for TE polarization and TM polarization, respectively. For the practical applications, the fabrication is imperfect. The actual parameters fabrication tolerances need to be discussed, which can influence the final performances. Table 1 shows the actual parameters fabrication tolerances with the period of 780 nm and duty cycle of 0.65 under Littrow mounting. In Table 1, the values of groove depth and thickness are in the vicinity of the optimal results, which can be a good guided mode to fabricate such the device. As can be seen from Table 1, efficiencies in the −1st and the 0th orders are more than 48.0% for TE and TM polarizations, where the corresponding different grating groove depth and thickness of connecting layer are obtained.

**Table 1:** The actual parameters fabrication tolerances with the period of 780 nm and duty cycle of 0.65 under Littrow mounting.

*h*_*g*_ (μm)	*h*_*c*_ (μm)	${{\boldsymbol{\eta }}}_{{\bf{0}}}^{{\bf{T}}{\bf{E}}}{\boldsymbol{(}}{\boldsymbol{ \% }}{\boldsymbol{)}}$	${{\boldsymbol{\eta }}}_{{\boldsymbol{-}}{\bf{1}}}^{{\bf{T}}{\bf{E}}}{\boldsymbol{(}}{\boldsymbol{ \% }}{\boldsymbol{)}}$	${{\boldsymbol{\eta }}}_{{\bf{0}}}^{{\bf{T}}{\bf{M}}}({\boldsymbol{ \% }}{\boldsymbol{)}}$	${{\boldsymbol{\eta }}}_{{\boldsymbol{-}}{\bf{1}}}^{{\bf{T}}{\bf{M}}}{\boldsymbol{(}}{\boldsymbol{ \% }}{\boldsymbol{)}}$
0.94	0.12	48.6	48.8	48.2	50.0
0.94	0.13	48.9	48.6	48.6	49.4
0.94	0.14	49.2	48.3	49.0	49.1
0.94	0.15	49.5	48.0	49.3	48.7
0.95	0.14	48.4	49.1	48.2	50.0
0.95	0.15	48.6	48.8	48.5	49.5
0.95	0.16	48.7	48.5	48.7	49.0
0.95	0.17	48.7	48.2	48.8	48.5

## Discussion

**Figure 4:**
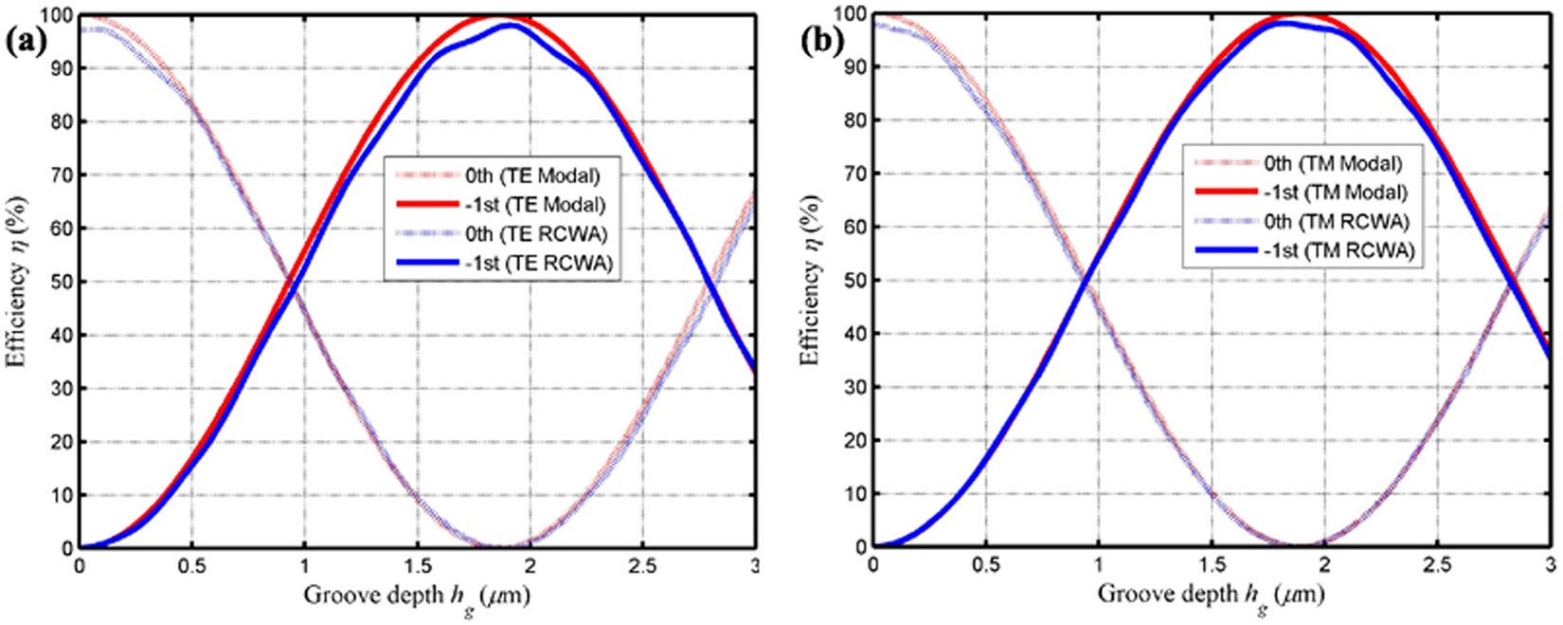
Diffraction efficiency versus groove depth in the 0th and the −1st orders based on simplified modal method and RCWA: (**a**) TE-polarized light and (**b**) TM-polarized light.

Figure 4 expresses the efficiency versus grating groove depth about TE and TM polarizations in the −1st and the 0th orders. In Fig. 4, the red curves based on the simplified modal method can be agreed with the blue curves on account of RCWA for both two-polarized lights. Because of neglecting the reflection of the grating propagating modes at the grating boundary surface, the diffraction efficiencies obtained by using simplified modal method are slightly larger than the numerical results obtained by employing RCWA.

## Methods

By means of the simplified modal theory, TE-polarized light or TM-polarized light is coupled into a propagating even mode and a propagating odd mode, the modes propagate inside the grating ridge with different effective indices. At the bottom of the connecting layer, the modes are coupled into the −1st and the 0th diffraction orders and the phase differences for TE and TM polarizations are accumulated, the phase differences of Δφ^TE^ and Δφ^TM^ can determine the diffraction efficiencies of the −1st and the 0th orders for two polarizations. Hence, the related equations are denoted by^16^:

1 $${\eta }_{0}^{TE}={\cos }^{2}(\frac{\Delta {\phi }^{TE}}{2}),{\eta }_{0}^{TM}={\cos }^{2}(\frac{\Delta {\phi }^{TM}}{2})$$

2 $${\eta }_{-1}^{TE}={\sin }^{2}(\frac{{\rm{\Delta }}{\phi }^{TE}}{2}),{\eta }_{-1}^{TM}={\sin }^{2}(\frac{\Delta {\phi }^{TM}}{2})$$

**Figure 5:**
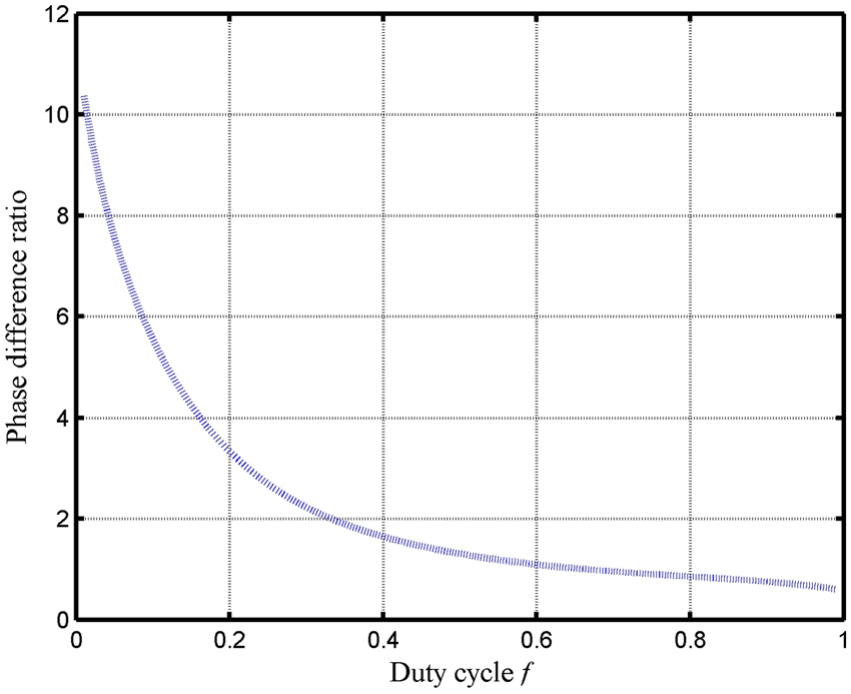
The phase difference ratio between Δφ^TE^ and Δφ^TM^ versus the grating duty cycle at an incident wavelength of 800 nm and a period of 780 nm.

**Figure 6:**
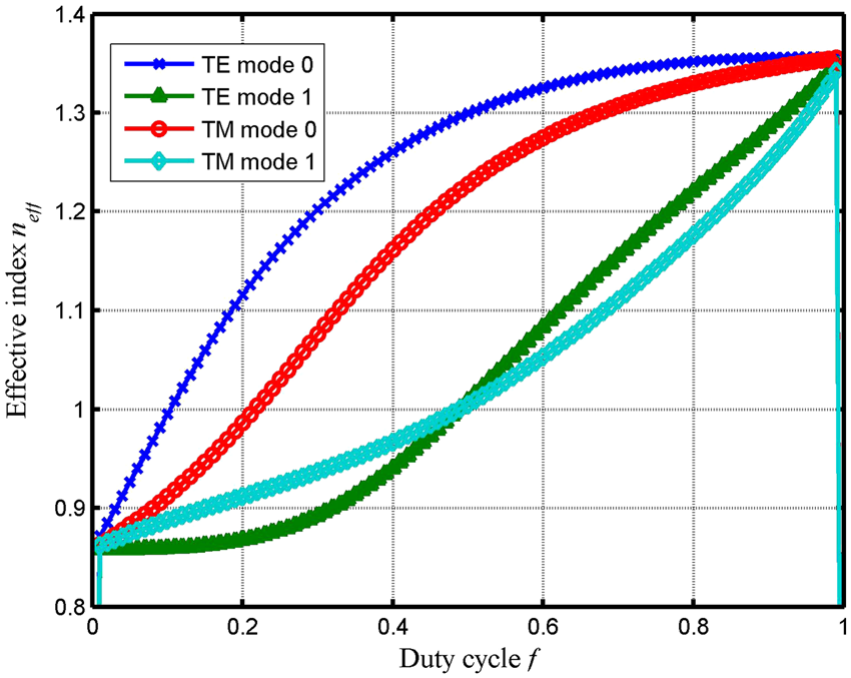
Effective index versus the grating duty cycle under the Littrow mounting.

As can be seen from Equations (1) and (2), in order to realize the 50/50 beam splitter grating, the powers in the −1st order and the 0th order should be equally distributed for TE and TM polarizations. Based on the simplified modal method, the accumulated phase differences of Δφ^TE^ and Δφ^TM^ should both meet odd-numbered multiples of π/2. Therefore, the expression of phase difference ratio is expressed by^16^:

3 $$\frac{\Delta {\phi }^{TE}}{\Delta {\phi }^{TM}}=\frac{\frac{2\pi }{\lambda }({n}_{0eff}^{TE}-{n}_{1eff}^{TE}){h}_{g}}{\frac{2\pi }{\lambda }({n}_{0eff}^{TM}-{n}_{1eff}^{TM}){h}_{g}}=\frac{2p+1}{2q+1}$$

where *n*_*veff*_^*TW*^ can be expressed as the *v*th effective indices corresponding *TW* polarization, *p* and *q* are both integers. In our practical etching procedure, *p* and *q* are set equal to zeros, which can save the etching material and simplify etching process. In order to obtain the optimal groove depth and thickness of connecting layer, we need to fix the grating period, which can be obtained by solving the grating equation:

4 $$\sin ({\theta }_{diff})=\frac{(2{n}_{2}m+1)\cdot \lambda }{2{n}_{2}d}$$

where the *θ*_*diff*_ is the diffraction angle, *m* is the diffraction order. In this design, we choose that the grating period is 780 nm. Figure 5 shows the phase difference ratio versus the grating duty cycle. As shown in Fig. 5, the curve is diminished within the duty cycle range of 0.00–1.00. At the wavelength of 800 nm based on simplified modal method, with the different grating duty cycle, the phase difference for TE and TM polarizations are different. Within the duty cycle range of 0.00–1.00, the phase difference for TE polarization can be gradually diminished, the phase difference for TM polarization can be gradually increased. At the point of duty cycle of 0.65, the value of Δφ^TE^/Δφ^TM^ is 1.015, which can be in close proximity to 1. Figure 6 depicts the effective index versus the grating duty cycle at wavelength of 800 nm and period of 780 nm. In Fig. 6, based on designed duty cycle of 0.65, the effective indices of TE polarization and TM polarizations are shown: ${n}_{0eff}^{TE}=1.3346,{n}_{1eff}^{TE}=1.1197,{n}_{0eff}^{TM}=1.2927,{n}_{1eff}^{TM}=1.0809$. In Fig. 6, with the duty cycle increased, the grating modes can propagate in the wider effective grating region, therefore the effective index for TE and TM polarizations are also increased.

**Figure 7:**
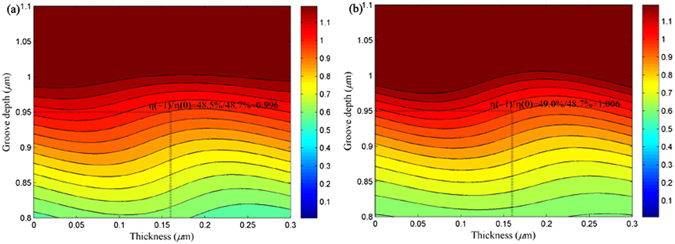
The grating groove depth and thickness versus the contour of the efficiency’s ratio between the −1st order and the 0th order: (**a**) TE-polarized light and (**b**) TM-polarized light.

By using simplified modal method, the grating duty cycle of 0.65 is confirmed. More importantly, it is necessary to evaluate grating groove depth, which can be easily optimized. For TE-polarized light, the grating groove depth of *h*_*g*_ is estimated that of 0.93 μm. For TM-polarized light, the *h*_*g*_ is estimated to be 0.94 μm. Thus, the exact groove depth must be approached to 0.93 μm or 0.94 μm. Based on the physical theoretical analyzation in the grating region according to the simplified modal method, the physical origin of this 50/50 beam splitter is that the output diffraction wave can propagate in the grating ridge and the different phase differences in the −1st order and the 0th order for TE and TM polarizations are accumulated. According to Equations (1) and (2), the approximated values of phase difference can be substituted into the equations and the estimated efficiencies in the −1st order and the 0th order for TE and TM polarizations are all almost closed to 50% with the duty cycle of 0.65 and period of 780 nm. With the certain duty cycle of 0.65 and period of 780 nm by means of simplified modal method, an accurate numerical simulated method of RCWA is an effective tool, which can resolve the boundary conditions for obtaining the precise diffraction efficiencies of the −1st and the 0th orders. During the numerical calculated precedure, the depth of *h*_*g*_ and thickness of *h*_*c*_ are searched and optimized. Figure 7 demonstrates the grating groove depth and thickness versus the diffraction efficiency’s ratios between the −1st order and the 0th order for both TE- and TM-polarized lights. In Fig. 7(a), the efficiency’s ratio is 0.996, where the TE-polarized light can diffract the efficiencies of 48.5% in the −1st order and 48.7% in the 0th order with the optimized depth of *h*_*g*_ = 0.95 μm and thickness of *h*_*c*_ = 0.16 μm. In Fig. 7(b), efficiencies in the −1st and the 0th orders can be separated into 49.0% and 48.7%, respectively for TM-polarized light along with the optimum depth and thickness under Littrow angle, where the efficiency’s ratio between the −1st order and the 0th order equals to 1.006.

### Conclusions

In this paper, a novel two-port transmission connecting-layer-based sandwiched grating is theoretically analyzed and numerically designed by using simplified modal method and RCWA, respectively. By use of the guided simplified modal approach, grating duty cycle of 0.65 is well designed and the evaluated grating groove depth can be guided to easily find the optimized depth of 0.95 μm. According to the optimized depth of 0.95 μm and thickness of 0.16 μm, the diffraction efficiencies calculated based on the simplified modal method and RCWA can be matched well with each other. A wide spectral bandwidth of 63 nm and a broad angular width of 9.57° for both TE and TM polarizations are obtained. Hence, the grating would be helpful in numerous photoelectricity integrated components.
